# Whole-body perfusion improves intraoperative transfusions in neonatal aortic arch surgery

**DOI:** 10.1093/icvts/ivad065

**Published:** 2023-05-12

**Authors:** Rodrigo Sandoval Boburg, Rafal Berger, Migdat Mustafi, Charlotte Faust, Harry Magunia, Felix Neunhoeffer, Michael Hofbeck, Peter Rosenberger, Christian Schlensak

**Affiliations:** Department of Thoracic and Cardiovascular Surgery, University Hospital Tübingen, Tübingen, Germany; Department of Thoracic and Cardiovascular Surgery, University Hospital Tübingen, Tübingen, Germany; Department of Thoracic and Cardiovascular Surgery, University Hospital Tübingen, Tübingen, Germany; Department of Anaesthesiology and Intensive Care Medicine, University Hospital Tübingen, Eberhard-Karls-University Tübingen, Tübingen, Germany; Department of Anaesthesiology and Intensive Care Medicine, University Hospital Tübingen, Eberhard-Karls-University Tübingen, Tübingen, Germany; Department of Pediatric Cardiology and Intensive Medicine, University Hospital Tübingen, Eberhard-Karls-University Tübingen, Tübingen, Germany; Department of Pediatric Cardiology and Intensive Medicine, University Hospital Tübingen, Eberhard-Karls-University Tübingen, Tübingen, Germany; Department of Anaesthesiology and Intensive Care Medicine, University Hospital Tübingen, Eberhard-Karls-University Tübingen, Tübingen, Germany; Department of Thoracic and Cardiovascular Surgery, University Hospital Tübingen, Tübingen, Germany

**Keywords:** Whole-body perfusion, Neonatal aortic arch surgery, Antegrade cerebral perfusion, Intraoperative transfusions

## Abstract

**OBJECTIVES:**

Whole-body perfusion is the combination of lower body perfusion and antegrade cerebral perfusion. This perfusion technique is used in some centres when performing aortic arch reconstruction surgery in neonates and infants. Several studies have shown intra- and postoperative benefits of this technique. However, no studies have analysed the impact it may have on the transfusion of blood products and coagulation factors.

**METHODS:**

We retrospectively analysed 65 consecutive neonates and infants who underwent aortic arch reconstruction surgery from January 2014 to July 2020. Patients operated from 2014 to 2017 underwent surgery with antegrade cerebral perfusion; in patients who underwent surgery from 2017 to 2020 a whole-body perfusion strategy was used. Demographic, intra- and postoperative parameters were compared as well as intraoperative blood product and coagulation factor transfusions, chest-tube output in the first 24 h and postoperative bleeding complications.

**RESULTS:**

Both groups required intraoperative transfusion of red blood cells, fresh frozen plasma, and platelets, as well as substitution of coagulation factors. The amount of transfused volumes of red blood cells, fresh frozen plasma and platelets (*P*-values 0.01, <0.01 and <0.01) and intraoperative transfusions of fibrinogen and von Willebrand factor were significantly lower in the whole-body perfusion group (*P*-value 0.04 and <0.01).

**CONCLUSIONS:**

A whole-body perfusion strategy may lead to fewer intraoperative blood product and coagulation factor transfusions when compared to antegrade cerebral perfusion alone in neonates and infants undergoing complex aortic arch reconstruction surgery.

**Figure:**
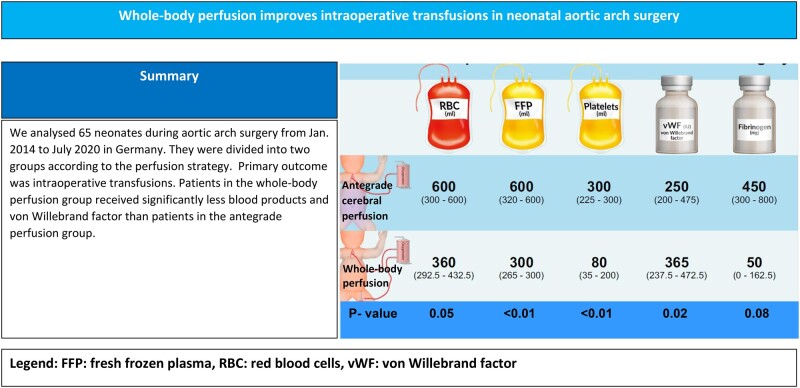

## INTRODUCTION

Aortic arch reconstruction surgery represents a challenge for the whole team involved in the treatment of these complex patients, especially in neonates and infants. In recent years there has been an increasing trend towards whole-body perfusion (WBP) therapy which entails antegrade cerebral perfusion (ACP) and lower body perfusion (LBP) during these types of surgeries [1–4]. Several studies have shown benefits from a WBP strategy as opposed to ACP [1–3].

However, no study has focused on the impact WBP may have on the intraoperative coagulation and the subsequent need for blood-product- and coagulation factor transfusions. It is well known that with the initiation of cardiopulmonary bypass (CPB) and subsequent blood contact with foreign materials (i.e. arterial- and venous lines) platelet activation and aggregation take place [5, 6]. This effect is directly related to the duration of CPB and the temperature at which it is performed, rendering hypothermia another factor which directly influences coagulation [7–9].

Nowadays, it is possible to perform thromboelastography (TEG) perioperatively as opposed to waiting for a coagulation blood work and acquire information regarding various coagulation factors which may be deranged and causing increased intraoperative bleeding [10–12]. With this information it is possible for anaesthesiologists to accurately substitute the lacking factors, i.e. fresh frozen plasma (FFP) and fibrinogen, among others.

Studies have shown that the need for perioperative transfusion of blood products as well as coagulation factors may be directly related to postoperative bleeding episodes and increased morbidity [13].

The objective of this study was to determine if the technique of WBP has an impact on the intraoperative bleeding based on the quantification of intraoperative blood-product transfusions and coagulation factor substitution as well as the incidence of re-do surgeries due to cardiac tamponade or postoperative bleeding compared to ACP.

## METHODS

### Ethical statement

This retrospective study was approved by the ethics committee of the Tübingen University Hospital (project number 461/2019BO2). Due to the retrospective nature of the study written consent of the parents was not necessary.

### Patient selection and grouping

**Figure 1:**
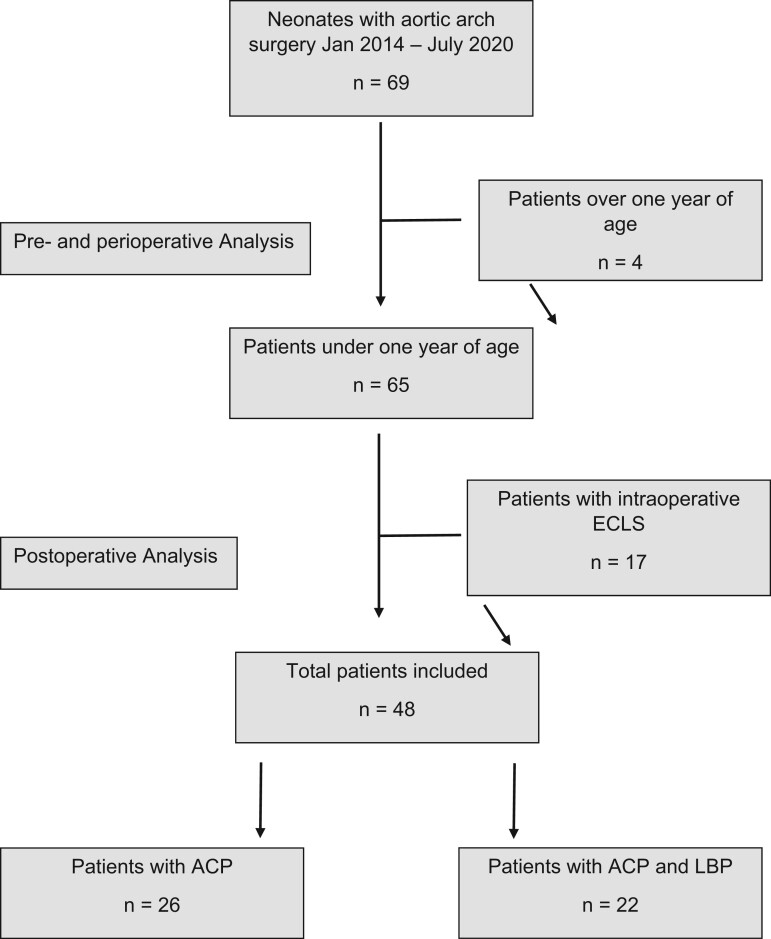
Patient selection chart. ACP: antegrade cerebral perfusion; LBP: lower body perfusion.

We retrospectively analysed all neonates and infants under 1 year of age who underwent aortic arch surgery at the Tübingen University Hospital from January 2014 to July 2020. Patients were included in this study if they underwent surgeries such as the Norwood procedure, Damus–Kaye–Stansel anastomoses or an aortic arch reconstruction due to hypoplasia as a primary-, part of a complex- or a re-do surgery. Figure 1 shows a patient selection chart. All procedures were performed by the same experienced surgeon and the same anaesthesiology team. Before January 2017, all patients undergoing any of these procedures were treated with ACP only. After January 2017, a WBP technique was established at our centre based on a review of the recent literature consisting of the standard ACP with additional LBP [4, 14]. All paediatric patients undergoing aortic arch surgery after this date were treated with WBP.

We included a total of 65 patients in the pre- and perioperative analysis, 34 in the ACP group and 31 in the WBP group. Seventeen patients who were dependent on postoperative extracorporeal life support (ECLS) were included for the pre- and intraoperative analysis up to the point in surgery where the decision to use an ECLS was taken; from that point on, patients were excluded, due to the greater number of intraoperative transfusions required for the ECLS implantation and in the postoperative phase. After exclusion the WBP group was made up of 22 patients and the ACP group of 26.

### Perfusion strategies

ACP was performed via a polytetrafluoroethylene shunt anastomosed to the brachiocephalic trunk where the arterial cannula was inserted. During ACP, cerebral perfusion was established at a rate of 50–80 ml/kg and monitored with near-infrared spectroscopy. For patients in the ACP group the target temperature for arch-repair was 28°C.

LBP was established following an ultrasound-guided percutaneous cannulation of the femoral artery with a 3 or 4 Fr arterial sheath; this was performed after anaesthesia induction by the team of experienced cardiac anaesthesiologists. This procedure had been previously described by our group [4]. The arterial sheath was connected to the CPB through an independent pump. During aortic clamping and aortic arch reconstruction combined ACP and LBP was established, the flowrate of LBP was 20-40ml/kg, this was measured by a Fumaflow Flowsensor (Fumedica, Muri, Switzerland) and adjusted depending on blood pressure, lactate levels, and peripheral oxygen saturation. The target temperature for these patients during aortic arch repair was moderate hypothermia at 30°C. After aortic arch reconstruction was completed conventional full-body perfusion was re-established.

In both groups the target peripheral oxygen saturation was >90%, lactate <3 mmol/l and mean arterial pressure 40 mmHg. If any of these parameters were not met with the current flow rate, it was adjusted and re-evaluated.

Following surgery patients were evaluated at least 3 times daily at the PICU for any signs of haematoma in the groyne, loss of pulses or limb ischaemia after removal of the femoral sheath.

### Transfusion regime

In all patients who weighed 5 kg or less, prior to starting CPB, the circuit was filled with 50–100 ml red blood cells (RBC) depending on preoperative haemoglobin levels and 50 ml FFP. Prior to termination of CPB, the patients were transfused with the contents of the CPB circuit. After rewarming and CPB termination, patients received a bolus dose of 25 IU/kg body weight of prothrombin-complex concentrate (PCC) and 50 mg fibrinogen and coagulation blood work was sent for analysis. During the first years of the study period ROTEM was utilized for coagulation analysis, in 2018 ROTEM was replaced by TEG for intraoperative coagulation monitoring. Arterial blood gas analysis was performed periodically with the purpose of controlling haemoglobin levels.

The clinical bleeding status together with ROTEM or TEG and coagulation blood work determined which coagulation products and factors were needed and respectively transfused. Factors VII and XIII were substituted only in cases of persistent bleeding despite normal blood work, normal TEG analysis and repeated transfusions. Starting in 2018, the use of von Willebrand factor (vWF) was restricted only to cases presenting with microvascular bleeding.

### Parameter analysis

We recorded demographic data such as gender, age, height, and weight at the time of surgery, and preoperative platelet count.

Intraoperative data included type of surgery, total duration of surgery (min), duration of CPB (min), duration of aortic cross-clamp (min) and the platelet count after termination of the CPB. We recorded intraoperative transfusions of blood products such as RBC (ml), FFP (ml), thrombocyte pools (TP) (ml) and intraoperative substitution of coagulation factors such as fibrinogen, vWF, PCC, factor XIII, and recombinant factor VII.

Postoperative data included documentation of bleeding episodes, re-do surgeries due to bleeding or acute pericardial tamponade, length of stay (LOS) at the paediatric intensive care unit (PICU), length of mechanical ventilation (MV) and chest-tube output (CTO) in the first 24 h.

### Statistical analysis

Statistical analyses were performed using the SSPS 23.0 (IBM Corporation, Armonk, NY, USA) software. Normal distribution was checked using the Kolmogorov-Smirnov test. Continuous variables were reported as mean and standard deviation if they fulfilled criteria of a normal distribution, otherwise median and interquartile ranges were calculated. Normally distributed variables were compared using the Student’s *t*-test and not normally distributed variables were compared using the Mann–Whitney *U*-test. Ordinal variables were reported as absolute values and percentages and were compared with the chi-squared test. Statistical analysis was performed in accordance with the guidelines of The European Journal of Cardiothoracic Surgery [15]. We reported our data according to the STROBE guidelines [16].

## RESULTS

There were no differences between both groups regarding age, weight, and gender. The majority of patients in this cohort were male. The types of surgical procedures are specified in Table 1. Intraoperatively, there was no difference between the groups regarding duration of surgery, CPB, or aortic cross-clamp time (Table 2). According to the different temperature strategy there was a significant difference between both groups regarding the lowest temperature during CPB. We compared the initial preoperative platelet count and found no difference between the groups (*P*-value 0.6). In addition, there was no difference between groups regarding the first platelet count following termination of CPB (*P*-value 0.5) and the possibility of primary chest closure (*P*-value 0.3) (Table 2).

**Table 1:** Demographic parameters and surgeries performed

	Antegrade cerebral perfusion, *N* = 34	Whole-body perfusion, *N* = 31	*P*-Value
Age (days), median (IQR)	13.5 (8–28)	14 (9–46)	0.3
Weight (kg), median (IQR)	3.4 (3.1–3.7)	3.7 (3.1–3.9)	0.3
Gender (male), *n* (%)	23 (67.6)	19 (61.3)	0.4
Surgeries performed, *n* (%)			
Norwood type	19 (55.9)	19 (61.3)	1
Aortic arch reconstruction	6 (17.6)	6 (19.3)	1
Aortic arch reconstruction, VSD and RV–PA conduit	4 (11.8)	1 (3.2)	0.7
Aortic arch reconstruction, ASD, VSD	2 (5.9)	1 (3.2)	1
Damus–Kaye–Stansel anastomoses and aortic arch	1 (2.9)	2 (6.5)	1
Arterial switch and aortic arch reconstruction	2 (8)	2 (6.5)	1

ASD: atrial septum defect; IQR: interquartie range; PA: pulmonary artery; RV: right ventricle; VSD: ventricular septum defect.

**Table 2:** Intraoperative parameters

	Antegrade cerebral perfusion, *N* = 34	Whole-body perfusion, *N* = 31	*P*-Value
Surgery duration (min) , median (IQR)	411 (353–510)	354 (293–551)	0.5
CPB duration (min), mean ± SD	174.8 ± 64.9	165.1 ± 66.3	0.3
Cross-clamp (min), mean ± SD	85.7 ± 47.9	90.9 ± 45.9	0.3
Lowest temperature (°C), median (IQR)	26.37 (25.8–28)	29 (28–30)	<0.01
Preoperative thrombocytes (1000/µl), median (IQR)	321 (237–415)	297 (232–402)	0.6
Intraoperative thrombocytes (1000/µl), median (IQR)	219.5 (157–280)	203 (169–340)	0.5
Primary chest closure, *n* (%)	12 (35.3)	15 (48.4)	0.3

CPB: cardiopulmonary bypass; IQR: interquartie range; SD: standard deviation.

We then analysed and compared the intraoperative transfusions between the groups. There was a significant difference in favour of the WBP group. As seen in Table 3, patients in the WBP group needed less RBC, FFP and TP (*P*-value 0.05, <0.01 and <0.01 respectively). The data in our cohort showed no difference regarding the platelet count between both groups. However, this result may have been influenced by the fact, that some patients in both groups received TP transfusions after termination of CPB before coagulation blood work was performed. The transfusion of intraoperative coagulation factors showed no significant difference in the amounts of recombinant factor VII, factor XIII, fibrinogen, and PCC (*P*-value 1, 0.7, 0.08 and 0.5). The amount of transfused vWF was significantly lower in the WBP group (*P*-value and <0.01) (Table 3).

**Table 3:** Intraoperative transfusions

	Antegrade cerebral perfusion, *N* = 34	Whole-body perfusion, *N* = 31	*P*-Value
RBC (ml), median (IQR)	600 (300–725)	360 (300–600)	0.05
FFP (ml), median (IQR)	600 (342.5–600)	300 (272.5–30)	<0.01
TP (ml), median (IQR)	300 (150–300)	60 (0–200)	<0.01
PCC (IU), median (IQR)	250 (37.5–550)	225 (200–350)	0.8
Factor XIII (IU), median (IQR)	0 (0–0)	0	0.69
Von Willebrand factor (IU), median (IQR)	250 (175–500)	150 (0–400)	0.02
Fibrinogen (mg), median (IQR)	500 (325–1000)	400 (250–550)	0.08
Factor VII (mg), median (IQR)	0 (0–0.3)	0 (0–0.3)	1

FFP: fresh frozen plasma; IU: international units; IQR: interquartie range; PCC: prothrombin complex concentrate; RBC: red blood cells; TP: thrombocyte pool.

Finally, we analysed the clinical parameters LOS at the PICU, length of MV and number of patients who suffered bleeding complications in the first 24 h after surgery (Table 4). While there was no difference regarding LOS, we found however a significant difference regarding the length of MV in favour of the WBP group (*P*-value 0.02). There were 3 patients in the ACP group who had to undergo surgical revision, 2 of them suffered an acute tamponade, versus 1 patient in the WBP group who underwent surgical revision because of pericardial haematoma. This difference proved to be statistically insignificant. There was no significant difference regarding the postoperative CTO during the first 24 h. There were no complications (i.e. haematoma, loss of pulses or limb ischaemia) related to the placement of the femoral artery sheath in patients of the WBP group.

**Table 4:** Postoperative parameters and bleeding events

	Antegrade cerebral perfusion, *N* = 26	Whole-body perfusion, *N* = 22	*P*-Value
LOS at PICU (days), median (IQR)	12 (8.8–22.5)	15 (7.75–14.8)	0.27
Length of MV (days), mean ± SD	7 ± 4.1	5.7 ± 3.6	0.02
Bleeding events (*n*), *n* (%)	3 (11.5)	1 (4.5)	0.6
Chest tube output (ml), median (IQR)	31 (18–62.3)	25 (20.3–50.5)	0.4

IQR: interquartile range; LOS: length of stay; MV: mechanical ventilation; PICU: paediatric intensive care unit; SD: standard deviation.

## DISCUSSION

In recent years, there has been an increasing interest in the technique of LBP when performing aortic arch reconstruction in neonates and infants. The studies, to this date, have focused mainly on postoperative organ function, especially the kidneys and the liver, and have been able to show benefits compared to ACP [1, 3, 17]. Fernández-Doblas *et al.* [3] showed that with LBP there is an improved liver function, in terms of rapid recovery of prothrombin time and internationalized ratio to normal levels; however, they did not study the impact this technique may have on intra- and postoperative coagulation as well as bleeding episodes. In a recent publication, which focused on postoperative outcomes neonates undergoing aortic arch surgery, we demonstrated, that patients with WBP had significantly less neurological complications than patients with ACP, most of these complications involved intracranial bleeding episodes [18]. To this date, there is no study in the literature that focuses on coagulation parameters in this group of patients.

Due to the low incidence of congenital lesions requiring extensive aortic arch surgeries, we can only report a cohort of 65 patients during the study period. As seen in Table 1, the groups compared were homogeneous regarding demographic parameters as well as the types of operations performed. Analysis of the intraoperative parameters showed no differences regarding duration of surgery, CPB or aortic cross-clamp time. The arterial sheath used for LBP was placed after the induction of anaesthesia by the cardiac anaesthesiologists; therefore, it had no influence on the length of surgery but might have lengthened anaesthesia induction time.

Unlike patients in the WBP group, patients in the ACP group had no direct LBP. Pigula *et al.* could prove in their comparative studies that during ACP, a certain degree of LBP takes place through collateral arteries, which may protect the abdominal organs against ischaemia [19, 20]. However, the work by Fernández-Doblas *et al.* showed a better postoperative hepatic function within the first 72 hours in patients supported with WBP, with close to normal internationalized ratio and prothrombin time values. From these results we can infer that with improved LBP, normalization of the coagulation after ending CPB is rapidly achieved and therefore less transfusions may be necessary. As seen in Table 2, patients in the WBP group of our study required significantly less RBC, FFP and TP transfusions. To the best of our knowledge there are no data in the literature comparing the requirement of intraoperative transfusions among different bypass strategies in infants. The work by Karimi *et al.* [21] reports the number of intraoperative RBC transfusions in paediatric cardiac surgery patients during 3 different time periods in the USA; unfortunately, the perfusion strategies during these time periods were not differentiated. However, they showed that neonates require the greatest number of intraoperative transfusions; in the neonatal group, patients with HLHS were leading with an average intraoperative of 3 units of RBC [21]. The lowest number of RBC transfusions (in the last recorded year 2014) in this report was higher than the average in our ACP group (2.7 vs 2 units) and even higher when compared to the WBP group (1 unit). Regarding the amount of RBC transfused, it is important to note, that a certain amount is used to fill the CPB, the amount is quantified and added to the total in the clinical documentation. Nevertheless, we can see that the median amount transfused in the ACP group (600 ml) almost doubles that of the WBP group (360 ml). We attribute the smaller amount of transfused RBC to the same reasons mentioned earlier.

After ending CPB, coagulation bloodwork was sent to the laboratory and ROTEM until 2018, then TEG diagnostic was performed to search for any coagulation factor deficiencies that may be substituted. Although there was a change in the device used to perform point-of-care analysis of the coagulation, studies have found, that the results between the automated ROTEM and TEG devices are similar and can both be utilized to identify coagulopathies due to surgery of bleeding [22]. We found no significant differences in PCC, factor XIII, fibrinogen and recombinant factor VII. Vitamin K-dependent coagulation factor levels were usually low and needed to be substituted in most of these patients. Recombinant factor VII and factor XIII were rarely substituted and were used only in situations of persistent bleeding despite the absence of TEG abnormalities.

We found a significant difference in the amount of vWF that were transfused, favouring the WBP group. vWF levels rise as an acute response to the inflammation caused by CPB and hypothermia [23]. After the initial rise in vWF these factors are consumed leading to an acquired vWF deficiency after termination of CPB, thus impairing coagulation [23]. Intraoperative acquired hypofibrinogenemia may be the result of haemodilution from CPB and plays an important role in intraoperative bleeding if not addressed adequately; however, this value was not significantly different between the groups [24].

Regarding the preoperative and first intraoperative platelet counts, we did not find any differences between both groups. However, it is important to note, that in some patients in both groups, an intraoperative blood sample was sent for analysis after transfusion of TP. Having this in mind, it is interesting, to see that the intraoperative platelet count of the ACP group, although not significant, was lower than the WBP group.

Analysis of the postoperative clinical parameters revealed no significant difference in the LOS at the PICU. Patients in the WBP group had a significantly shorter MV time; this may be in part due to a certain degree of volume overload caused by the intraoperative transfusions required in the ACP group, although there may have been other factors contributing to this difference [25].

Regarding the postoperative bleeding events and complications, there was no significant difference concerning the CTO during the first 24 h between both groups. Interestingly, the significant difference in the intraoperative transfusion of coagulation factors and blood products did not translate into clinical benefit or fewer bleeding episodes: There were 3 bleeding events in the ACP group, 2 of which were acute pericardial tamponades due to coagulopathy requiring immediate evacuation; the last one was a pericardial haematoma diagnosed in a routine echocardiography which was evacuated during mediastinal exploration. In the WBP group, there was only 1 patient with pericardial haematoma who was diagnosed in a routine echocardiography and was also evacuated during a mediastinal exploration. These data are in accordance with Zwifelhofer *et al.* [26] who reported that a deranged intraoperative coagulation does not directly correlate with excessive postoperative bleeding.

A factor that may have played a significant role in our study is the difference in target temperatures in both groups. It is well known that the degree and duration of hypothermia progressively impair clotting time, speed of clot formation, and maximal clot strength as measured by TEG [7, 11]. Therefore, the higher target temperature in the WBP group may have had a positive effect on the intraoperative bleeding tendency in this group. In our opinion, it is difficult to differentiate the possible effects of the higher target temperature from the benefits of improved abdominal perfusion on the coagulation of these patients. However, LBP provided the opportunity to perform the complex surgical procedures under mild, as opposed to moderate, hypothermia. Irrespective of the share of contribution of the separate influences, the combination of both components had a beneficial effect on intraoperative bleeding and requirement of intraoperative transfusions.

### Limitations

Due to the relatively low incidence of severe aortic arch hypoplasia, our single-centre study covers a long time period to collect a significant number of patients. During this time period there was both a change in the anaesthesia protocol, regarding the use of vWF and a change in the intraoperative testing, from ROTEM to TEG in 2018. We cannot exclude that these factors might have had some influence on the results of our study. We intend to perform a prospective multicentre study based on a larger number of patients to confirm our results.

## CONCLUSION

In neonates and infants undergoing aortic arch reconstruction surgery, a WBP strategy may lead to a reduction of intraoperative blood product and coagulation factor transfusion when compared to ACP. Nevertheless, in our cohort, the need for less transfusions did not translate to significantly fewer surgical explorations due to postoperative bleeding or higher CTO.

**Conflict of interest:** Harry Magunia received a speaker honorary from CSL Behring. The other authors report no conflict of interest.

## Data Availability

The data used in this manuscript cannot be made publicly available due to patient-specific data. However, it can be put at your disposal upon written request to the corresponding author. **Rodrigo Sandoval Boburg:** Conceptualization; Data curation; Formal analysis; Methodology; Validation; Visualization; Writing—original draft; Writing—review & editing. **Rafal Berger:** Conceptualization; Data curation; Validation; Writing—review & editing. **Migdat Mustafi:** Conceptualization; Methodology; Validation; Writing—review & editing. **Charlotte Faust:** Conceptualization; Investigation; Validation; Writing—original draft; Writing—review & editing. **Harry Magunia:** Conceptualization; Methodology; Validation; Writing—review & editing. **Felix Neunhoeffer:** Conceptualization; Methodology; Validation; Writing—review & editing. **Michael Hofbeck:** Conceptualization; Investigation; Supervision; Validation; Writing—original draft; Writing—review & editing. **Peter Rosenberger:** Conceptualization; Writing—review & editing. **Christian Schlensak:** Conceptualization; Methodology; Supervision; Writing—original draft; Writing—review & editing. Interactive CardioVascular and Thoracic Surgery thanks David Kalfa and the other anonymous reviewer(s) for their contribution to the peer review process of this article.

## ABBREVIATIONS

ACP: Antegrade cerebral perfusion
CPB: Cardiopulmonary bypass
CTO: Chest-tube output
ECLS: Extracorporeal life support
FFP: Fresh frozen plasma
LBP: Lower body perfusion
MV: Mechanical ventilation
PCC: Prothrombin-complex concentrate
PICU: Paediatric intensive care unit
RBC: Red blood cells
TEG: Thromboelastography
TP: Thrombocyte pools
vWF: von Willebrand factor
WBP: Whole-body perfusion
